# Smart, Tunable CQDs with Antioxidant Properties for Biomedical Applications—Ecofriendly Synthesis and Characterization

**DOI:** 10.3390/molecules25030736

**Published:** 2020-02-08

**Authors:** Łukasz Janus, Julia Radwan-Pragłowska, Marek Piątkowski, Dariusz Bogdał

**Affiliations:** Department of Biotechnology and Physical Chemistry, Faculty of Chemical Engineering and Technology, Cracow University of Technology, Warszawska 24 Street, 31-155 Cracow, Poland; lukasz.janus@doktorant.pk.edu.pl (Ł.J.); j.radwan@doktorant.pk.edu.pl (J.R.-P.); pcbogdal@cyf-kr.edu.pl (D.B.)

**Keywords:** carbon dots synthesis, surface modification, fluorescent nanomaterials

## Abstract

Carbon quantum dots (CQDs) are nanoobjects of a size below 10 nm. Due to their favorable features, such as tunable luminescence, unique optical properties, water solubility, and lack of cytotoxicity, they are willingly applied in biomedicine. They can be obtained via bottom-up and top-down methods. However, to increase their quantum yield they must undergo post-processing. The aim of the following research was to obtain a new type of CQDs modified with a rhodamine b derivative to enhance their fluorescence performance without biocompability deterioration. For their preparation glucose was used as a precursor and four different carbonizing agents which affected semi- and final products luminescence properties. The ready nanomaterials were investigated over their chemical structure by FTIR and NMR, whereas morphology was investigated by the TEM method. Their optical properties were determined by UV–VIS spectroscopy. Fluorescence behavior, photo- and pH-stability, as well as solvatochromism showed their applicability in various biomedical applications due to the controlled properties. The samples exhibited excellent antioxidant activity and lack of cytotoxicity on L929 mouse fibroblasts. The results showed that proposed strategy enables preparation of the superior nanomaterials with outstanding luminescence properties such as quantum yield up to 17% which can be successfully applied in cell labelling, bioimaging, and theranostics.

## 1. Introduction

Recent decades have shown that nanomaterials constitute a powerful tool in numerous industrial branches [1]. A promising tool are quantum dots (QDs) which can be described as objects of the size below 10 nm with unique properties [1,2,3,4]. Since their development, they have been successfully used in optoelectronics [1], food packaging [5], metals detection [6,7,8], bioimaging [9,10,11,12,13,14], photocatalysis [15,16], sensing [17,18,19,20,21,22,23], cell labelling [24], or fluorescent inks and others [25,26]. The first-generation quantum dots are semiconductors of crystalline structure [3]. In general, quantum dots have narrow emission spectra and broad absorption [3,27]. The most common ones are CdSe/ZnS, CdSeS/ZnS, and CdTe/ZnS, which were used in various biological applications due to their superiority over conventional fluorescent dyes, such as high photostability or excited state lifetime. Thanks to the aforementioned properties, QDs could be applied in long-term cell labelling and for dynamic biochemical process tracking [27]. Quantum dots were also successfully used in multicolor imaging due to their unique feature: an ability to be excited by one light source and detect different colors simultaneously [3]. However, these QDs were found to be toxic since they may generate reactive oxygen species (ROS) which are known to damage various proteins, lipids, and genetic material [27,28]. Moreover, Cd-based nanomaterials can release cadmium ions leading to cell death. Importantly, it occurred that quantum dots may undergo certain modifications resulting in the change of their surface or chemical structure [27,28]. Finally, their preparation is expensive. Therefore, there was a need for a novel type of quantum dots which would be suitable for biomedical applications. In 2004, a new type of carbon-based materials was accidentally obtained during nanotube purification [29]. Until then, numerous types of carbon quantum dots (CQDs) have been obtained with various possible applications due to their very attractive properties, such as luminesce, good water solubility, high photostability, resistance to photobleaching or photoblinking, possibility, chemical inertness and, finally, lack of toxicity and biocompability [1,2,3]. Furthermore, CQDs’ region of photoluminescence is very broad, starting in the ultraviolet and ending in the near-infrared. Although, until now, the luminescence behavior of carbon nanodots is still not fully clear, it is believed that crucial role plays two factors, namely, that the quantum size is below 10 nm and surface defects which may act as excitation energy traps. Interestingly, CQDs can successfully act as both electron donors and acceptors [2,3,4].

CQDs can be prepared from various resources, such as peels (mango, orange, banana, pineapple, onion, seaweed etc.), meat, grains, nuts, or vegetable by-products, which makes them a low-cost material [30,31]. Currently, these nanomaterials are widely applied in drug delivery, gene delivery, biosensing, bioimaging, cell labelling, and many other fields, including optoelectronics, photovoltaics, and photocatalysis [24,25,26]. There are two main methods for carbon quantum dot obtainment, namely, bottom-up and top-down, which differ in terms of the carbon source [30,31]. Pure carbon has low water solubility and weak fluorescence. Top-down preparation routes include electrochemical oxidation, laser ablation, and ultrasound. Of note, they require harsh synthesis conditions and the obtainment pathway can be complex. These methods allow control over the nanostructure and size of the resulting nanomaterials. For example, it is possible to obtain nanodots 2–3 nm in size using C60 fullerenes as the starting material [24]. However, the so called “one step” approaches enable the preparation of carbon dots with mediocre biocompability and unsatisfying quantum yield. Therefore, in most cases they are followed with the “second step” which is surface passivation or modification [30,31]. The bottom-up approach, on the other hand, uses simple raw materials, such as glucose, aminoacid, fresh vegetables, or waste biomass. In fact, most of the organic compounds rich in carbon atoms can be applied as a feedstock. Thus, currently, the most desirable CQD preparation methods are solvothermal and hydrothermal ones due to their ecofriendly nature and possibility of carbon dot self-passivation during the synthesis [2,32]. Aforementioned CQD obtainment pathways are inexpensive, fast, and scalable [30,31]. Nevertheless, they also have some major drawbacks, such as poor control over CQD size, which is extremely important in the case of biomedical applications. This obstacle can be defeated by post-treatment, such as dialysis, sonification, electrophoresis, or column chromatography [24].

Surface modification is a major issue regardless future CQD applications since it may enhance quantum yield, increase photostability, or enable interaction with certain drugs, biomolecules, and proteins, cells, and tissues [24]. Noteworthy, the post-processing method must be adjusted to CQDs’ potential use, such as cell labelling or bioimaging, and such factors as the type of the raw material used or the synthesis method must be taken under consideration. A very popular approach is the application of polymers as modifying agents, which can be poly(anions), poly(cations), and neutral, depending on the specified needs [24,33,34,35]. Polyethylene glycol enables preparation of CQDs which will not cause immune response, whereas positively-charged polyethyleneimine enables obtainment of nanodots capable of cell membrane integrity disruption resulting in more efficient cell transfection. The aforementioned polymer also enables CQD binding with DNA and RNA and turns raw nanodots into thermo- or pH-sensitive nanomaterials. Neutral or negatively-charged macromolecules are used for the CQDs’ surface modification with therapeutic designation since they prevent protein adsorption, resulting in prolonged circulation in blood [24]. There are also numerous scientific reports regarding other ways of carbon quantum dot functionalization, including doping and combining with other types of nanoparticles [21,36,37,38,39]. Application of heteroatoms, such as nitrogen and sulfur, helps to create new surface states which results in enhanced photoluminescence (PL) properties, especially increased photostability and quantum yield [10,24,35]. This can be achieved using l-cysteine combined with citric acid as CQD starters. The carbon dots doped with nitrogen atoms were reported to enrich a quantum yield (QY) of 22% in ethanol and exhibited good biocompatability. It has been reported that CQDs combined with silica nanoparticles resulted in an increase of specificity and selectively in drug delivery applications. Preparation of the more complex nanostructures may result in the obtainment of a powerful tool for cancer treatment, such as a simple system—theranostic agent or a more complex one, namely, a bioplatform [24]. For example, the combination of triphenylphosphonium with mesoporous silica magnetite resulted in the preparation of a nanomaterial which could be applied in cell imaging, mitochondrial targeting and, finally, magnetic field improved cellular uptake. Similar functionalization of the carbon quantum dots was carried out using hyaluronic acid which enabled controlled doxorubicine delivery coupled with real-time bioimaging [24]. Aforementioned examples show that novel approaches of carbon quantum dot functionalization are highly desired since, although there are still many issues to be overcome, CQD-based nanomaterials may play a crucial role in the development of nanomedicine [2,36,37,38,39].

The aim of the following research was to develop a new type of carbon quantum dots functionalized with novel rhodamine derivatives to enhance their PL. The nanomaterials were prepared using glucose as a carbon source. Raw CQDs, as well as ready products, were investigated over their chemical structure, morphology, and spectroscopic properties. Their luminescence behavior depending on pH and solvent type was determined. Additionally, the antioxidant activity of the CQDs has been studied, as well as cytotoxicity on the mouse fibroblasts L929 cell line. The results showed that the proposed approach resulted in the preparation of nanomaterials with superior, tunable PL characteristics followed by good photostability and biocompability. Novel types of carbon quantum dots with modified surfaces may play an important role in the development of smart luminescence systems for medicine and pharmacy, especially in the area of cancer detection and treatment.

## 2. Results

### 2.1. FourierTransform Infrared Spectroscopy (FTIR) and Nuclear Magnetic Resonanse (NMR) Analysis of the Modified Carbon Quantum Dots

**Scheme 1:**
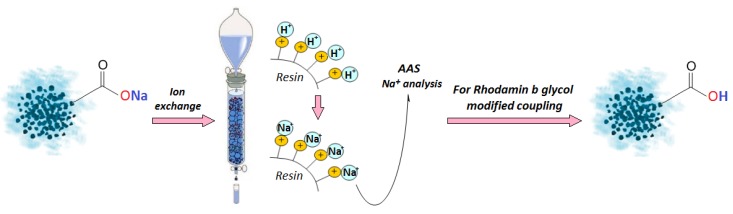
Carbon quantum dots with the free carboxylic group preparation pathway.

The surface modification is a well-known approach to enhance quantum dots properties. However, formation of chemical bonds between functional groups of the CQDs with the reactive groups of the modifying agent can be challenging. To prepare carbon dots for surface modification, electrostatically, sodium ions were removed leaving free carboxyl groups (Scheme 1).

**Figure 1:**
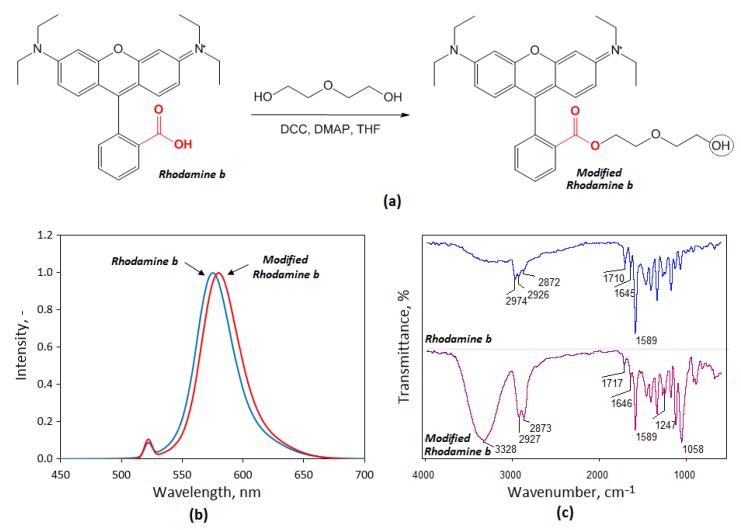
(**a**) Rhodamine b modification pathway; (**b**) fluorescence spectra of the rhodamine b and modified rhodamine; (**c**) FTIR of the rhodamine b and its derivative, *N*-(9-(2-carboxyphenyl)-6-(diethylamino)-3*H*-xanthen-3-ylidene)-*N*-ethylethanaminium (Rhod-OH).

**Figure 2:**
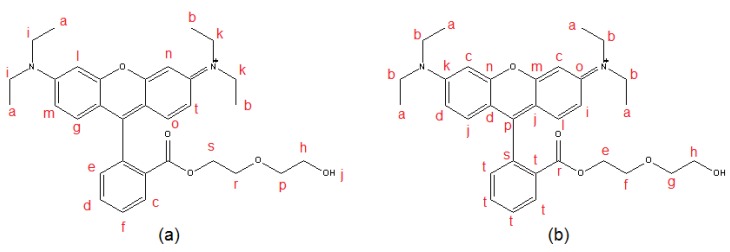
Chemical structure of the *N*-(9-(2-carboxyphenyl)-6-(diethylamino)-3*H*-xanthen-3-ylidene)-*N*-ethylethanaminium (Rhod-OH): (**a**) ^1^H-NMR; (**b**) ^13^C-NMR.

Rhodamine, which is known to be a non-toxic fluorescent dye, has a high potential in bioimaging due to its satisfying quantum yield [40,41,42]. However, its chemical structure, apart from conjugated unsaturated bonds responsible for its luminescent behavior, contains one functional group capable of coupling with other substance. Thus, it was converted into a hydroxyl one through diethylene glycol grafting, as shown in Figure 1a. The incorporation of the glycol was confirmed by UV–VIS analysis (Figure 1b) which display a 5 nm shift to the higher wavelengths compared to the native dye. FTIR analysis presented in Figure 1c shows some changes in the rhodamine b spectrum, such as the newly formed band coming from hydroxyl groups at 3328 cm^−1^ and 1058 cm^−1^, as well as a band of the increased intensity corresponding to acyl chain (2927 cm^−1^ and 2873 cm^−1^). The chemical linkage formation between hydroxyl groups from diethylene glycol and rhodamine carboxyl groups proves the arisen band at 1717 cm^−1^, which is a characteristic for ester bonds. The resulting derivative—Rhod-OH—was further investigated over its chemical structure by ^1^H-NMR and ^13^C-NMR methods (Figure 2). The chemical shifts presented in Table 1 correspond to the data obtained from FTIR analysis and clearly confirm that the *N*-(9-(2-carboxyphenyl)-6-(diethylamino)-3*H*-xanthen-3-ylidene)-*N*-ethylethanaminium is a product of rhodamine b modification. The Rhod-OH was used for carbon quantum dot modification.

**Figure 3:**
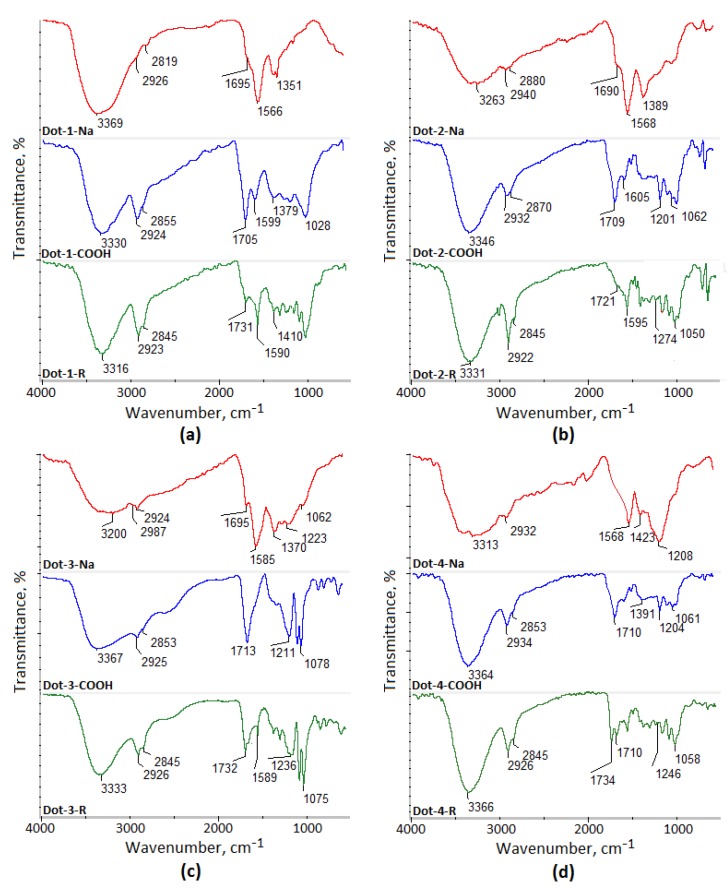
FTIR spectra of the raw CQDs, CQDs with free carboxyl groups and modified CQDs (**a**) Dot-1-Na (Dot-1 sample containing sodium ions), Dot-1-COOH (sample with removed sodium ions and unblocked free carboxylic groups), Dot-1-R (dots modified with Rhod-OH); (**b**) Dot-2-Na (Dot-2 sample containing sodium ions), Dot-2-COOH (sample with removed sodium ions and unblocked free carboxylic groups), Dot-2-R (dots modified with Rhod-OH); (**c**) Dot-1-Na (Dot-3 sample containing sodium ions), Dot-3-COOH (sample with removed sodium ions and unblocked free carboxylic groups), Dot-3-R (dots modified with Rhod-OH); (**d**) Dot-4-Na (Dot-4 sample containing sodium ions), Dot-4-COOH (sample with removed sodium ions and unblocked free carboxylic groups), Dot-4-R (dots modified with Rhod-OH).

Figure 3 presents the FTIR spectra of the raw carbon quantum dots, carbon quantum dots with unblocked carboxylic groups, and Rhod-OH modified quantum dots. Carbon dots (Dot-1) given in Figure 3a were prepared using glucose as a precursor and formic acid as a carbonizing agent via hydrothermal reaction. Their spectrum (red) shows a band coming from OH and NH stretching vibrations at 3369 at cm^−1^, bands of low intensity typical for -CH- groups at 2926 cm^−1^ and 2819 cm^−1^. Moreover, a slightly shifted band results from the free carboxylic groups at 1695 cm^−1^, a band of high intensity can be assigned to the sodium salt at 1566 cm^−1^, and a band results from the C-H stretching at 1351 cm^−1^. The Dot-1-COOH spectrum (blue) shows that the sodium ion removal process was successful since the band of high intensity at 1705 cm^−1^, which corresponds to carboxyl groups, is present and a very wide band with the maximum at 3320 cm^−1^ is also present. Importantly, a band at 1599 cm^−1^ coming from C=C is visible. Conjugated unsaturated bonds are responsible for the luminescence properties, thus, their presence is desirable. Generally, all of the FTIR spectra are typical for quantum dots [43,44]. The spectrum of Dot-1-R shows carbon quantum dots modified with Rhod-OH. The successful modification is proved by the formation of ester bonds which are visible at 1731 cm^−1^. The Dot-2 FTIR spectra are presented in Figure 3b. The CQDs were prepared using aspartic acid as a carbonizing agent. The raw CQDs spectrum is similar to Dot-2-Na since analogous bands are visible like the one at 3263 cm^−1^ coming from free hydroxyl and amino groups, 2940 cm^−1^ and 2880 cm^−1^ which come from acyl groups. This band is of higher intensity compared to the Dot-1 sample, which is caused by the application of a carbonizing agent containing more carbon atoms in its chain. A band from free COOH groups is present at 1690 cm^−1^, whereas a band comes from free NH_2_ and sodium salt at 1568 cm^−1^. The aforementioned band has a higher intensity compared to Dot-1 due to the incorporation of amino groups coming from aspartic acid. Additionally, a band corresponding to C-H stretching is again visible at 1389 cm^−1^. The FTIR spectrum of Dot-2-COOH show the chemical structure of the dots after sodium ion removal, whose elimination confirms the band at 1709 cm^−1^ of high intensity as well as a band at 3346 cm^−1^. Finally, the Dot-2-R spectrum shows Rhod-OH-modified dots, which confirms the presence of a band typical for ester groups at 1721 cm^−1^. The sample prepared using tartaric acid (Dot-3) exhibits a similar chemical structure to previous dots as the same bands are observed. However, in this case the intensity of free carboxyl groups is lower than in previous samples both before and after modification. The last set of spectra are for the Dot-4 sample prepared using hydrochloric acid as a carbonizing agent. Herein, the FTIR spectrum contains the lowest number of various bands due to the least complicated structure of the acid. Again, in this case the electrostatically-bonded Na^+^ ions were successfully removed, and free carboxylic groups are present. As in the previous cases, the modification with Rhod-OH can be proved by the formation of ester bonds (1734 cm^−1^). What is important is that all final samples contain hydrophilic functional groups (OH, NH_2_, and COOH) as well as conjugated unsaturated bonds responsible for fluorescence behaviors. Nevertheless, it can also be noticed that the number of free groups is higher in the case of samples where an organic acid was used for the carbonization. The presence of hydrophilic groups, such as hydroxyl, carboxyl, and amino groups, is responsible for the excellent water solubility of the prepared CQDs. The results correspond to other researcher’s data [6,8,43,44].

### 2.2. Morphology Study

**Figure 4:**
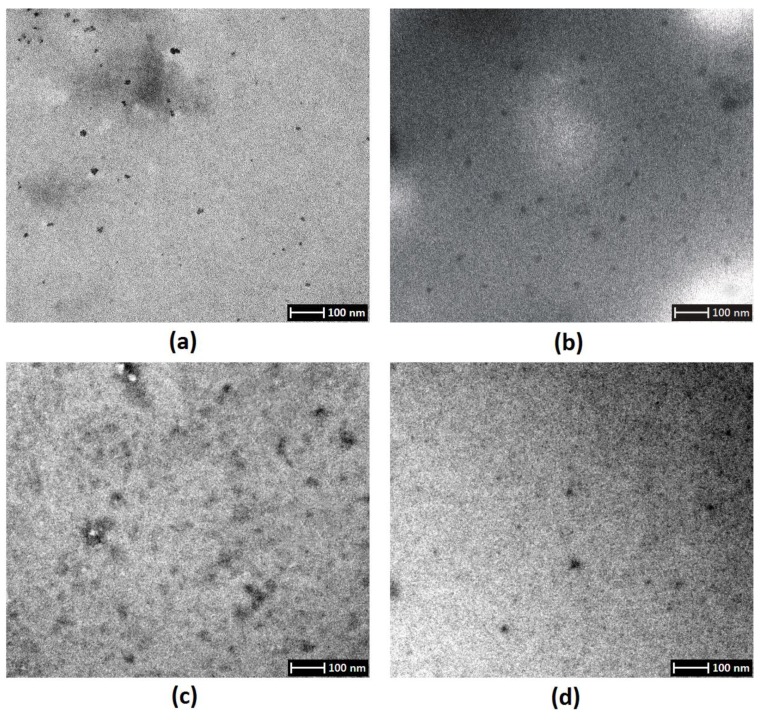
TEM images (**a**) Dot-1 sample; (**b**) Dot-2 sample; (**c**) Dot-3 sample; (**d**) Dot-4 sample.

Figure 4 shows TEM images which confirm the carbon nanodots preparation. It can be noticed that different carbonizing agents applied for the glucose carbonization (Table 2) caused diversification in their size and shape. However, their round-shaped morphology is typical for CQDs obtained by the bottom-up approach and corresponds to other researchers’ results [36,37,38,39,44]. It can be observed that the Dot-3 sample prepared using tartaric acid is the most uniform one and the average carbon dot diameter is 10 nm. The Dot-2 sample prepared using aspartic acid contains carbon dots of a similar character. The smallest particles were obtained using formic acid during the synthesis (sample Dot-1) with the size ranging from 3 nm to 10 nm. The quantum dots prepared using hydrochloric acid appear to be the of a polydispersive nature and have the highest range of size, which is from 2 nm to 11 nm. It has been reported that the CQDs’ size affects its luminescence behavior [1,2,3]. Thus, it can be assumed that the right choice of the carbonizing agent enables the preparation of nanodots with desired properties using the hydrothermal method, which is known to be challenging. The average size below 10 nm suggests that quantum dots should be characterized by good fluorescence and an ability to penetrate cell membranes [24,25,26].

### 2.3. Spectroscopic Properties Study

**Figure 5:**
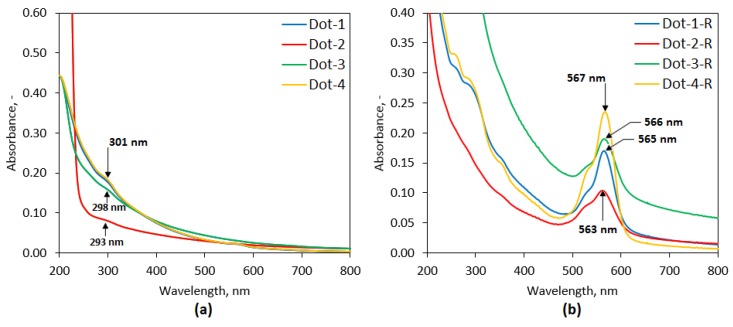
(**a**) UV–VIS spectra of the prepared CQDs from glucose; (**b**) UV–VIS spectra of the CQDs modified with Rhod-OH.

**Figure 6:**
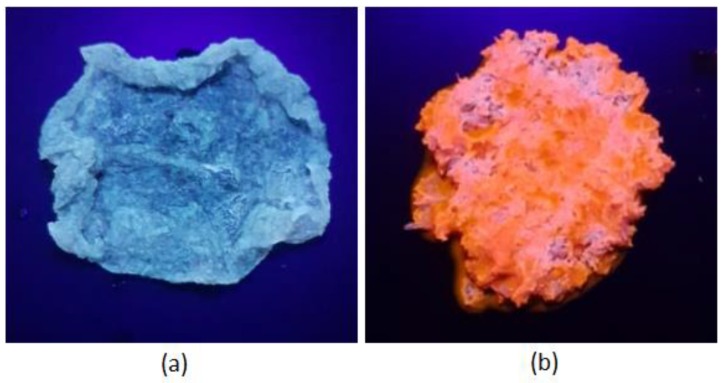
Chitosan sponges saturated with CQDs solutions visualized under UV light (**a**) Dot-1 sample; (**b**) Dot-1-R sample.

Figure 5 presents the optical properties of the prepared samples investigated by UV–VIS spectroscopy. Unmodified CQDs UV–VIS spectra are given in Figure 5a. It can be noticed that there are two strong absorption peaks present in the diluted CQD solutions, one sharp and one broad. The first one, around 200 nm, comes from the π → π* transitions of C=C groups which are caused by the sp2 hybridization of the C atoms [2], whereas the peaks at 293 nm (Dot-2), 298 nm (Dot-3), 301 nm (Dot-1), and 301 nm (Dot-4) corresponds to n → π* transitions of C=O groups [2,38,39,43]. The UV–VIS spectrum is typical for carbon quantum dots and two different surface states can be distinguished which agrees with the data obtained by other researchers [2,6,8,43]. The UV–VIS results correspond to the FTIR spectra (Figure 3) which showed bands typical for unsaturated carbon-carbon bonds as well as free carboxylic groups. It can be noticed that the spectra of samples Dot-1, Dot-3, and Dot-4 are similar, whereas Dot-2 is different, which can be caused by the presence of free amino groups coming from the aspartic acid, which was used as a carbonizing agent. Figure 5b shows UV–VIS spectra of the quantum dots coupled with rhodamine derivative. It can be noticed that the spectra are different than in the case of the raw CQDs (Figure 5a) since new peaks are present around 560 nm, which are typical for rhodamines and which confirms successful modification and agrees with the data present in the FTIR spectra. The raw dots have a brownish color under daylight, whereas under UV it changes to blue (365 nm). On the other hand, samples modified with Rhod-OH have a pink color which becomes orange under UV (Figure 6).

**Scheme 2:**
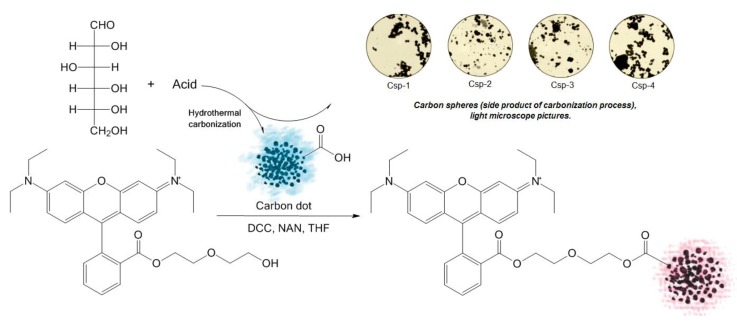
Surface-modified carbon quantum dot synthesis route.

**Figure 7:**
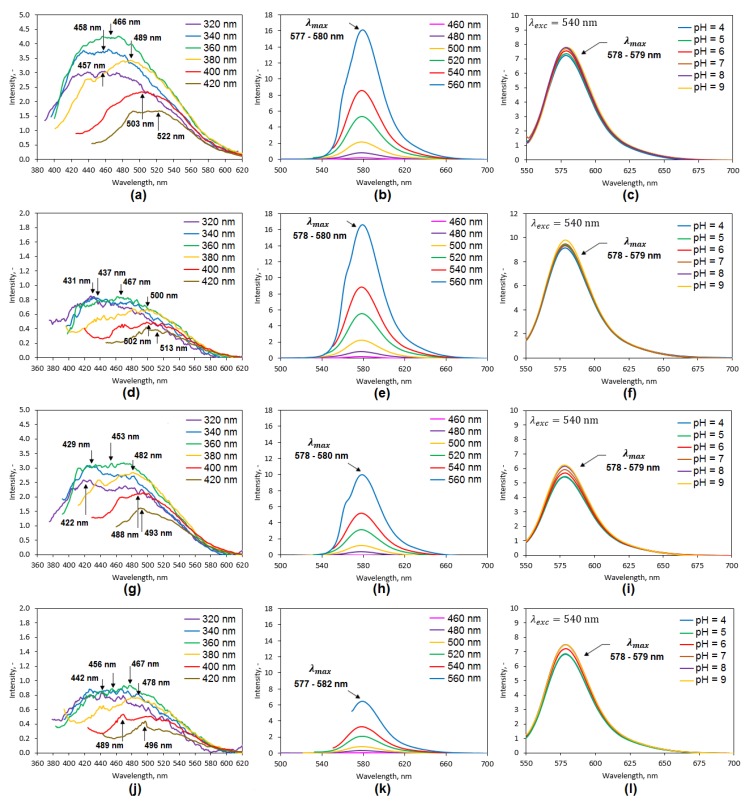
PL spectra of the prepared samples: (**a**) Dot-1 fluorescence spectrum; (**b**) Dot-R fluorescence spectrum; (**c**) Dot-1-R fluorescence dependence on pH; (**d**) Dot-2 fluorescence spectrum; (**e**) Dot-2-R fluorescence spectrum; (**f**) Dot-2-R fluorescence dependence on pH; (**g**) Dot-3 fluorescence spectrum; (**h**) Dot-3-R fluorescence spectrum; (**i**) Dot-3-R fluorescence dependence on pH; (**j**) Dot-4 fluorescence spectrum; (**k**) Dot-4-R fluorescence spectrum; (**l**) Dot-4-R fluorescence dependence on pH.

Figure 7 shows PL spectra of the obtained samples and their luminescence dependence on the pH values. Fluorescence spectra of the raw carbon dots (a: Dot-1; d: Dot-2; g: Dot-3; j: Dot-4) are typical for these quantum nanomaterials [2,4,6,8,37,38,43,44]. There is an obvious emission dependence on the visible excitation wavelength, which is typical for quantum nanobojects below 10 nm in size due to the surface defects, conjugated π-domains, as well as crystalline structure disturbance [1,2,3]. One may observe that the choice of the carbonizing agent has a significant impact on the PL properties, which proves different PL intensity of the unmodified CQDs. The highest intensity was spotted for Dot-1 sample prepared using formic acid which resulted in the formation of the CQDs with the lower size. Similar performance was obtained for the Dot-3 sample carbonized using tartaric acid, which contains hydroxyl and carboxyl groups. Dot-2 and Dot-4 samples have very similar PL characteristics which may be attributed to resembling the morphology of the nanodots which is known to determine various quantum effects [2]. The mediocre characteristics of Dot-2 corresponds to the UV–VIS results (Figure 4a). Fluorescence spectra of the modified samples using Rhod-OH are given in Figure 6 (b: Dot-1-R; e: Dot-2-R; h: Dot-3-R; k: Dot-4). The successful modification is once more confirmed since, just like in Figure 4b, the spectra exhibit significantly different characteristics. Importantly, the modified samples have the same maximum peak at approximately 580 nm, which is typical for rhodamine dyes. Additionally, the peaks are shifted to higher wavelengths. However, it can be noticed that the luminescence effects have a rather molecular, not quantum, nature which is caused by the efficient incorporation of Rhod-OH molecules into the carbon quantum dots. Thus, the PL performance can be attributed to the presence of conjugated unsaturated bonds and chromophores (Scheme 2) rather than quantum effects. What is interesting is that the choice of the carbonizing agent played a crucial role in the superficial modification since, depending on the acid used, different PL intensities are observed. The amount of the incorporated dye was different for each sample, namely Dot-1-R: 11.3 mg per g, Dot-2-R: 14.2 mg per g, Dot-3-R: 8.7 mg per g, and Dot-R-4: 3.9 mg per g. For the PL intensity in the case of the Dot-3 sample, the increase is the lowest (3×), while in the case of the Dot-2 sample there was an increase of almost 20 times. For the Dot-1 sample the PL intensity increased four times, and for Dot-4, around seven times. On the contrary to the unmodified samples, the highest fluorescence is for the Dot-2-R sample. In the case of all the Rhod-OH-modified CQDs, the highest PL intensity is observed for 560 nm excitation. In the case of 540 nm, the intensity decreases two times, whereas for wavelengths below 500 nm, the intensity increase is below 1, which suggests that the sample’s quantum effect is very poor, and the range of nanomaterials applicability is between 560–500 nm, which is much narrower than in the case of unmodified dots (320–420). Nevertheless, the range of 560–500 nm is commonly used in biomedical research [38,39,40], thus, it can be concluded that the best type of the nanodots will depend on the potential application. The nanomaterials were also investigated over their behavior under different pH conditions, which is an important feature in terms of biomedical applications since pH-sensitivity may sometimes disturb certain measurements and biochemical process investigations. Figure 7c,f,i,l presents modified CQD behaviors in various pHs, ranging from 4 to 9. It can be noticed that the nanomaterial behavior is not fully uniform. Samples Dot-1-R and Dot-2-R do not exhibit any pH dependence, whereas, in the case of Dot-3-R and Dot-4-R nanodots, the PL intensity decreases at lower pH values and slight luminescence quenching is observed, which is a desirable in some cases [23,24,25,26].

**Figure 8:**
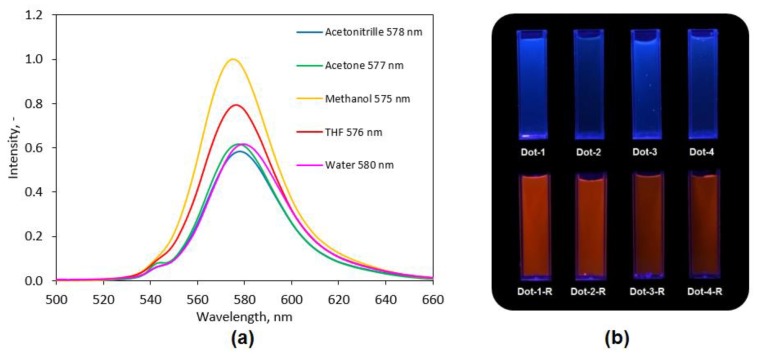
(**a**) Solvatochromism of the surface-modified carbon quantum dot (Dot-2-R); (**b**) prepared samples under UV light (365 nm) dissolved in distilled water.

One of the most interesting phenomena in the case of fluorescence dyes and nanomaterials is solvatochromism, which is an important feature in terms of biological applications [3]. Figure 8 shows the results of the solvatochromism study on Rhod-OH-modified Dot-2-R performed using five different solvents of different polarity, namely, acetone, acetonitrile, methanol, tetrahydrofuran, and water. It can be observed that PL intensity depends on the solvent and highest value is obtained for methanol, which corresponds to other researchers’ data [3]. The parameter is almost twice lower for water, acetone, and ACN, and a very small shift of the peak maximum is noticeable. Thus, it can be concluded that the nanomaterials may be applied in various applications and media of various hydrophility, which is important in the field of diagnostics, and from this point of view is a desirable feature [17,18,19,20,21,22,23].

Our previous research showed that the bottom-up CQD synthesis routes with in situ doping enables the preparation of nanodots with the maximal QY up to 13%, which may be insufficient in some applications [44]. Thus, a novel CQD modification method was developed which resulted in the preparation of the carbon materials with enhanced quantum yield (Table 2). One may observe that the QY of the unmodified carbon dots oscillated below 4%, which is typical for glucose precursors. The worst results were obtained for the Dot-4 sample which was prepared using hydrochloric acid. What is important is that all samples were thoroughly purified from post-reaction residues which are also known to have luminescence properties and which PL may be wrongly assigned to CQDs [44]. The highest QY was obtained for the Dot-1 sample prepared using tartaric acid (3.9%), probably due to the carboxylic and hydroxyl groups in its structure and Dot-1 (3.4%) obtained using formic acid. The quantum yield of the CQDs prepared by Chen et al. [43] using waste tea as a precursor was around 7.1%, which is much lower than newly developed surface-modified carbon dots. Tang et al. [3] prepared CQDs with a QY of 22%, however, in ethanol, not water, thus, in an aquatic environment their performance may be unsatisfactory for biological applications. The data given in Table 2 shows that the proposed modification method enabled significant improvement of CQDs QY, which is higher 10× around after coupling with Rhod-OH. The best results were obtained for the Dot-2-R sample (QY = 17%) which corresponds to data given in Figure 7. The worst QY (4.9%) was for Dot-4-R. All samples were additionally investigated over their photostability after seven and 30 days. High photo-stability is a desired feature, especially in long-term visualization and biochemical process investigation applications [3,24,25,26]. The results given in Table 2 show that all samples have excellent stability after 30 days, and the decrease in QY is barely noticeable and may be caused by an auto-passivation process or slight degradation.

### 2.4. Antioxidant Activity Study

Quantum dots, due to their extraordinary properties, have a great application in medicine and pharmacy, especially in cell labelling and long-term bioimaging. However, these nanomaterials have a tendency for reactive oxygen species generation [2,11,27,28,43] which negatively affects cell viability and biochemical processes, thus, significantly interrupting real-time investigations [2,27,28,43,44]. ROS cause lipid peroxidation, proteis, nucleic acid, or carbohydrate damage, and oxidative stress, which leads to cell apoptosis or necrosis as a consequence of metabolism product oxidation. During the following study, eight different types of CQD nanomaterials were developed. Their ability for free radical scavenging is presented in Table 3. One may observe that all of the samples exhibit excellent antioxidant properties in contact with hydroxyl and peroxide free radicals. The highest antioxidant activity was observed for the Dot-2 sample which was capable of removing 90% of OH^•^ and 92% of O_2_^•−^. The performance of Dot-3 was similar (88% OH^•^ and 90% O_2_^•−^ removed). The worst results were obtained in the case of unmodified CQDs for the Dot-1 sample prepared using formic acid, namely 75% (OH^•^) and 78% (O_2_^•−^). Great antioxidant properties can be assigned to the presence of functional groups capable of free radical neutralization as well as glucopyranose rings from glucose residues. In the case of samples modified with Rhod-OH the results are similar. Again, the sample prepared using aspartic acid (Dot-2-R) exhibits the best antioxidant properties (84% OH^•^ and 87% O_2_^•−^ removed) which leads to the conclusion that free amino groups take part in hydroxyl and superoxide radical neutralization. In all cases superoxide radicals appear to be easier to remove due to their lower resonance stability than hydroxyl ones.

### 2.5. Cytotoxicity Study

**Figure 9:**
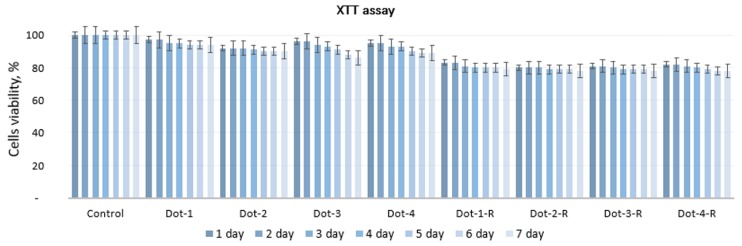
Results of cytotoxicity study (XTT assay).

Nanomaterials have numerous applications in various industrial branches. Their arising importance is due to unique physicochemical and biological properties which gives new detection and treatment possibilities, providing a powerful tool in biomedical areas [2,3,24]. Quantum dots, due to their PL characteristics, are willingly used in tissue engineering and bioimaging. However, to be applied for such purposes they cannot exhibit cytotoxicity, thus, Cd-based QDs are no longer approved [27,28]. Figure 9 shows the results of the cytotoxicity study carried out on L929 mouse fibroblasts which are typically used for biomaterial evaluations [44] for seven days with daily cells viability determination. It can be noticed that all samples do not exhibit cytotoxicity, even after seven days of continuous cell culture. However, small differences in biological behavior may be observed, namely, fibroblasts cultured in the presence of CQDs after modification with Rhod-OH have a slightly lower cell viability. On the other hand, the number of viable cells is more stable than in the case of fibroblasts cultured with pristine CQDs, which can be explained by their passivation or reaction with cell growth medium components. The highest change was noticed in the case of the Dot-3 sample, which may be caused by the oxidation of its functional groups. Nevertheless, this effect is negligible. Overall, the obtained data shows that all samples meet the basic requirements for biomedical applications according to ISO regulations for biomaterials, since none of them exhibited lethal activity to fibroblasts and, in any case, the cells’ viability was not below 70%.

## 3. Materials and Methods

### 3.1. Materials

Glucose, NaOH, HCl, FeSO_4_, NaCl, tetrahydrofuran, methanol, ethanol, acetone, acetonitrile, formic acid, and tartaric acid were purchased from POCH, Gliwice, Poland. L-aspartic acid, Tris-HCl, sodium acetate, diethylene glycol (DEG), *N*,*N*’-dicyclohexylcarbodiimide (DCC), 4-dimethylaminopyridine, pyrogallol, rhodamine b, XTT assay, mouse fibroblasts (L929 cell line), Dulbecco’s Modified Eagle Medium DMEM, streptomycin/penicillin (10%), trypsin, and PBS were purchased from Sigma–Aldrich, Poznań, Poland. Molecular weight cut off 500–1000 Da dialyzing membranes and 0.22 μm filter membranes were purchased from Bionovo, Zielona Góra, Poland. All reagents were of analytical grade purity.

### 3.2. Methods

#### 3.2.1. CQDs Preparation and Modification

Carbon quantum dots were prepared by a facile hydrothermal method using an autoclave reactor (150 mL). As a carbon source, glucose was applied. To obtain CQDs the organic precursor was mixed with water and the acid followed, being placed in the sealed vessel. The synthesis details are given in Table 4. The ready product was neutralized using NaOH until pH = 7. Next, the samples were purified from unreacted residues and macromolecular compounds via dialysis, which was carried out for five days (MWCO 500–1000 Da). Then, the amount of sodium ions present in the CQD structure was determined by atomic absorption spectroscopy (AAS) using a Philips PU9100x spectrometer (Melbourne, Australia). Next, the samples were analyzed by FTIR spectroscopy which showed a high content of sodium ions that were electrostatically bonded to the superficial carboxylic groups of the dots. To remove sodium ions and obtain free COOH groups on the CQDs, a surface cation-exchange column was applied (Scheme 1) which was followed by another FTIR analysis showing the disappearance of the band coming from Na salt and an increase of the signal corresponding to free carboxylic groups.

To perform surface modification a new rhodamine b derivative was obtained. For this purpose, rhodamine b was modified with diethylene glycol. The derivative was prepared by placing 0.5 g of the dye in 10 mL of tetrahydrofuran (THF), 4 mL of diethylene glycol (DEG), and 0.24 g of *N*,*N*’-dicyclohexylcarbodiimide (DCC). As a catalyst 4-dimethylaminopyridine (DMAP) was applied (0.05 g). The reaction was carried out under continuous stirring at room temperature for 24 h. The product was dried and dissolved in THF and purified using silica gel column chromatography. To determine the chemical structure of the Rhod-OH, NMR analyses were carried out. To perform ^1^H-NMR and ^13^C-NMR, 50 mg of the Rhod-OH was dissolved in deuterium-containing methanol and analyzed using FT-NMR 500 MHz JEOL system equipped with a Royal HFX probe (Warsaw, Poland). The proton NMR was carried out performing 16 scans, while carbon NMR involved 1024 scans.

To perform CQD coupling with (*N*-(9-(2-carboxyphenyl)-6-(diethylamino)-3*H*-xanthen-3-ylidene)-*N* ethylethanaminium) (Rhod-OH), 3 mL of carbon dots aquatic solution was used (5 mg/mL) which were further dissolved in 6 mL of THF. To the CQD solution, 15 mg of the Rhod-OH were added and 0.05 g of DCC. The reaction took place under room temperature and atmospheric pressure (24 h with constant stirring). The resulting product was dried to remove the solvents. The final CQDs were purified using MWCO dialysis membranes. The efficiency of the CQD modification was determined through the basic hydrolysis with 0.1 M NaOH solution followed by fluorescence measurement using a Jasco FP-750 spectrofluorometer.

#### 3.2.2. Optical Properties Study

UV–VIS analysis was carried out using an Agilent 8453 diode array spectrophotometer [44]. Fluorescence study was carried out using a Jasco FP-750 spectrofluorometer (JASCO, Tokio, Japan). Quantum yield was determined by the comparative method described in our previous article [44]. The study PL-pH dependence was carried out at pH = 4, 5, 6, 7, 8, 9. The pH was adjusted using an Elmetron CX-551 pH-meter (combined glass pH electrode, Elmetron, Zabrze, Poland). The solvatochromism study was carried out using the following solvents: water, acetone, acetonitrile, tetrahydrofuran, and methanol. Infrared analysis was performed using a Nexus 470 diamond crystal ATR FTIR spectrophotometer (Thermo Fisher Scientific (Waltham, MA, USA).

#### 3.2.3. Antioxidant Study

The antioxidant properties were investigated using two different types of free radicals, namely, superoxide and hydroxyl, according to the procedure described by Chen et al. [43]. Superoxide radical removal efficiency was determined using the autoxidation of pyrogallol method, while hydroxyl ones were determined by the Fenton reaction. To calculate the ability of free radical scavenging (S), the following equations were used:

S_S_, % = [(A_S_ − A_0_)/A_0_] × 100% (1)

where:

S_S_—scavenging ability to remove superoxide radicals, %;

A_0_—standard absorbance, -;

A_S_—sample absorbance, -.

S_OH_, % = [(A_S_ − A_0_)/(A − A_0_)] × 100% (2)

where:

S_OH_—scavenging ability to remove hydroxyl radicals, %;

A_0_—standard absorbance;

A_S_—Sample absorbance;

A—Solution without FeSO_4_ absorbance.

#### 3.2.4. Cytotoxicity Study

Tests of the cytotoxicity were carried out using L929 mouse fibroblasts by XTT assay. The cell proliferation was investigated after 24 h, 48 h, 72 h, 96 h, 120 h, 144 h, and 168 h (seven days). The cell culture was performed under standard conditions described in [44]. Before the tests, all solutions were sterilized using 0.2 µm membrane filters (Bionovo, Legnica, Poland). The cells were investigated under an inverted optical microscope Delta Optical (Zielona Góra, Poland).

## 4. Conclusions

In this study, a novel approach to carbon quantum dot (CQD) surface modification is presented. Nanodots were prepared using glucose as a precursor followed by coupling with a rhodamine derivative, which were characterized over their chemical structure and morphology. The resulting products exhibited enhanced fluorescence properties and superior quantum yield in water (17%). The nanomaterials after modification were characterized by a narrower emission spectrum shifted to longer wavelengths which is a highly desirable feature in terms of biological uses. The nanoobjects were proved to have superior antioxidant activity and were found to be non-toxic to L929 cells after seven days. The products exhibited excellent photostability and tunable properties due to their solvatochromism and adjustable pH behavior, which give them a broad spectrum of future applications in the area of medicine and pharmacy, including bioimaging, cell labelling, and theranostics.

## Figures and Tables

**Table 1:** NMR analysis of the rhodamine b derivative *N*-(9-(2-carboxyphenyl)-6-(diethylamino)-3*H*-xanthen-3-ylidene)-*N*-ethylethanaminium (Rhod-OH).

^1^H-NMR	Chemical Shift, ppm	^13^C-NMR	Chemical Shift, ppm
a	1.27	a	11.4
b	1.27	b	45.5
c	8.33	c	95.6
d	7.85	d	114.1
e	7.79	e	64.4
f	7.78	f	68.3
g	7.40	g	72.1
h	3.33	h	60.6
i	3.35	i	113.5
j	3.95	j	132.7
k	4.10	k	158.0
l	6.97	l	131.1
m	7.09	m	156.7
n	6.89	n	158.8
o	7.01	o	132.5
p	3.37	p	131.0
r	3.56	r	165.3
s	4.10	s	132.6
t	7.08	t	131.0

**Table 2:** Quantum yield determined in distilled water of the prepared CQDs and its photostability.

Sample	Quantum Yield, %	Quantum Yield After Seven Days, %	Quantum Yield After 30 Days, %
Rhod-OH	31	28	22
Dot-1	3.4	3.2	3.0
Dot-2	1.1	1.1	1.0
Dot-3	3.9	3.6	3.5
Dot-4	0.9	0.9	0.9
Dot-1-R	14.1	14.1	14.0
Dot-2-R	17.0	16.9	16.8
Dot-3-R	11.2	11.2	11.0
Dot-4-R	4.9	4.9	4.9

**Table 3:** Antioxidant properties of the prepared samples.

Sample	% of OH^•^ Free Radicals Removed	% of O_2_^•^^−^ Free Radicals Removed
Dot-1	75	78
Dot-2	90	92
Dot-3	88	90
Dot-4	80	83
Dot-1-R	81	83
Dot-2-R	84	87
Dot-3-R	82	85
Dot-4-R	82	84

**Table 4:** CQDs synthesis parameters.

Sample	Glucose g per 20 mL H_2_O	Acid, Amount	Reaction Time, h	Temperature, °C
Dot-1	9	Formic, 3.0 mL	12	180
Dot-2		Aspartic, 1.5 g		
Dot-3		Tartaric, 1.5 g		
Dot-4		Hydrochloric, 1.5 mL		
